# Comparison of different drug regimens for the treatment of loiasis—A TropNet retrospective study

**DOI:** 10.1371/journal.pntd.0006917

**Published:** 2018-11-01

**Authors:** Federico Gobbi, Emmanuel Bottieau, Olivier Bouchaud, Dora Buonfrate, Fernando Salvador, Gerardo Rojo-Marcos, Paola Rodari, Jan Clerinx, Begoña Treviño, Juan Paulo Herrera-Ávila, Andreas Neumayr, Guido Calleri, Andrea Angheben, Camilla Rothe, Lorenzo Zammarchi, Massimo Guerriero, Zeno Bisoffi

**Affiliations:** 1 IRCCS Center for Tropical Diseases, Ospedale Sacro Cuore Don Calabria, Negrar, Verona, Italy; 2 Department of Clinical Sciences, Institute of Tropical Medicine, Antwerp, Belgium; 3 Service des Maladies Infectieuses et Tropicales, CHU Avicenne, Assistance Publique Hôpitaux de Paris, Paris, France; 4 Infectious Diseases Department, Vall d’Hebron University Hospital, PROSICS Barcelona, Barcelona, Spain; 5 Hospital Universitario Príncipe de Asturias, Alcalá de Henares, Madrid, Spain; 6 International Health Unit Vall d’Hebron-Drassanes, PROSICS Barcelona, Barcelona, Spain; 7 Swiss Tropical and Public Health Institute, Basel, Switzerland; 8 Unit A of Infectious and Tropical Diseases, Amedeo di Savoia Hospital, Turin, Italy; 9 University Hospital Medical Centre, Hamburg, Germany; 10 LMU Medical Centre, Munich, Germany; 11 Dipartimento di Medicina Sperimentale e Clinica, Università di Firenze; SOD Malattie Infettive e Tropicali, Azienda Ospedaliero Universitaria Careggi, Florence, Italy; 12 Department of Computer Science, University of Verona, Verona, Italy; McGill University, CANADA

## Abstract

**Background:**

*Loa loa* infection is endemic in limited areas of West-Central Africa. Loiasis has been associated with excess mortality, but clinical studies on its treatment are scant, particularly outside endemic areas, due to the rarity of cases diagnosed.

**Methodology/Principal findings:**

With this retrospective TropNet (European Network for Tropical Medicine and Travel Health) study, we aimed at outlining the treatment schedules followed by different reference centers for tropical medicine across Europe. We gathered information about 238 cases of loiasis, 165 of which had follow up data. The regimens followed by the different centers were heterogeneous. The drugs most frequently administered were: diethylcarbamazine alone (74/165, 45.1%), ivermectin alone (41/165, 25%), albendazole + ivermectin (21/164, 11.6%), ivermectin + diethylcarbamazine (16/165, 9.7%).

**Conclusions/Significance:**

The management of loiasis substantially differs across specialized travel clinics in Europe. These discrepancies could be due to different local protocols as well as to (un)availability of the drugs. An harmonization of clinical protocols for the treatment of loiasis would be suggested across reference centers for tropical medicine in Europe.

## Author summary

*Loa loa* is a worm which infects millions of people living in wide forested areas of central Africa. The infection is rarely diagnosed outside Africa, and cases are mainly referred to referral centers on tropical medicine. Aim of this study was to describe the treatment and management of patients diagnosed with loiasis in some referral centers for tropical medicine in Europe. The results showed that different health centers treat the patients with different drugs/drug combinations. On the basis of the availability of the drugs and on the data observed, common protocols might be recommended for the participating centers.

## Introduction

*Loa loa* is a filarial worm transmitted to the human hosts by the tabanid flies of the genus *Chrysops*. Human loiasis occurs only in Africa, where the transmission is confined to the rainforests areas from south-eastern Benin in the west, South Sudan and Uganda in the east, North Angola in the south[1]. Adult worms moving in subcutaneous tissues can live for more than 15 years: moreover, after a prepatent period of a minimum of six months, they can produce microfilariae (mff), circulating in peripheral blood with a diurnal periodicity[2]. The most common clinical manifestations due to adult worms and/or mff are "Calabar swelling" (transient angioedema of allergic nature) and pruritus. Moreover, adult worms may be noticed when they pass under the conjunctiva of the eye ("eyeworm"). Albeit rarely, loiasis can cause damage to other organs[2]. Loiasis had been considered as a benign disease until a retrospective study recently demonstrated an excess of mortality in patients with high microfilaremia[3], and advocated more studies on this disease[4]. Currently three drugs may be used for the treatment of loiasis: diethylcarbamazine (DEC), ivermectin (IVM) and albendazole (ALB). DEC is the drug of choice because of its macro- and microfilaricidal activity, that causes a rapid decrease in the *Loa loa* microfilaremia, although sometimes multiple courses of DEC are required to achieve clinical and parasitological cure[5]. DEC is considered as contra-indicated in patients with a high microfilarial density (>8.000 mff/ml), because of the risk of encephalopathy[6]. Moreover, this drug is not currently available in Europe[7]. In fact, in an inquiry involving 69 TropNet (European Network for Tropical Medicine and Travel Health) centers in Europe, DEC was immediately available in 25 centers, available within a few days in 11, and not available in 33[8]. Ivermectin has a marked microfilaricidal effect (*Loa* microfilaremia decreases by 70–80% within the first 3 days after a single dose of 150 μg/kg)[9], but is probably not active on macrofilariae[6]. Besides this, this drug should be administered with caution in case of microfilaremia > 8,000/ml and can also induce an encephalopathy in people with very high microfilarial densities (>30,000/ml), contra-indicating its use above that level[6,10]. Short courses of ALB have little effect on *Loa loa*[11,12], but when given at a dose of 200 mg twice a day for 21 days, the drug has probably an embryotoxic effect (i.e., it interrupts embryogenesis in the uterus of the adult female worm), and possibly also a macrofilaricidal effect[13]. This being said, the treatment strategy depends firstly on the risk of adverse events, which is related to the patient's *Loa* microfilarial density. In fact, given the risk of serious adverse events after DEC or IVM treatment, Boussinesq proposed the following strategy: ALB for microfilarial loads higher than 8,000/mL, followed by IVM when microfilarial density is between 2,000 and 8,000/mL, DEC when microfilarial density is below 2,000/mL[6]. Two series published in 1986[14] and in 1996[15], reported that a single course of treatment with DEC for 21 days achieves a cure rate of 55% and 66%, respectively. Four recent papers, a case series of 47 imported cases in France[16], a review of 101 cases reported in non-endemic countries[17], a case series of 100 cases in a single centre in Italy[18], and a case series of 50 cases in London[19], revealed a wide heterogeneity of treatment regimens and follow -up patterns over the last three decades across Europe, highlighting that the management of imported loiasis needs standardization. A randomized controlled trial (RCT) comparing different drugs in non-endemic countries, is problematic, due to the relatively small number of cases diagnosed per travel clinic and the differences in drug availability between countries. The primary objective of this study was to describe the different drugs regimens used for imported loiasis in different TropNet sites. Secondary objectives include the description of the treatment outcome and tolerability of the drugs used.

## Methods

This is an observational, retrospective study, analyzing data collected between 1996 and 2015 at TropNet centers participating in this study.

### Ethical issues

The study protocol was submitted to the Ethics committee of the coordinating centre (Comitato Etico per la Sperimentazione Clinica delle Province di Verona e Rovigo), and obtained a waiver of informed consent on the 13^th^ July 2016.

### Inclusion criteria

All individuals who had been traveling or living in an endemic country AND were diagnosed with loiasis (according to the case definition given below) AND were treated with either DEC alone, IVM alone, ALB alone, ALB + DEC, IVM + DEC or ALB + IVM (at the dosages specified below) AND had at least one follow up visit ≥ 1 month after treatment AND had not traveled to any endemic country before the last follow up evaluation. Eligible drug regimens for patient inclusion: DEC, 6 mg/kg/day for 21 days; IVM, 150–200 μg/kg as a single dose; ALB, 200–400 mg twice a day for 21–28 days.

### Exclusion criteria

All patients treated with any other drug regimen. Patients retreated after first treatment failure.

### Definitions

A case of loiasis was defined in this study as the presence -or history (in the last two months)—of eyeworm OR the demonstration of *Loa loa* microfilaremia OR the presence -or history (in the last two months)- of a Calabar swelling associated with eosinophilia (defined as > 450 eosinophils/μL).

Clearance of symptoms was defined as the lack of re-occurrence of the clinical signs/symptoms present at inclusion (Calabar swellings, eyeworm) in the time frame going from treatment to the last available follow up visit. Parasitological outcome was assessed on the basis of values of microfilaremia and eosinophil count at the last available follow up visit. Circulating microfilariae were detected in 9 mL of peripheral blood collected on daytime using a modified Knott technique followed by Giemsa staining for species identification.

For each patient, information on clinical history, laboratory examinations, and treatment was extracted from the medical records, and entered into a Google Drive case report form (CRF). An anonymous code was assigned to each patient.

### Primary outcome

1. Frequency of drug regimens used to treat imported loiasis.

### Secondary outcomes

1. Proportion of patients with negativization of microfilaremia and normal eosinophil count at the last available follow-up for each treatment, over the total number of patients who received that treatment.
2. Proportion of patients presenting clearance of symptoms for each treatment strategy, over the total number of patients receiving that treatment.
3. Trend in residual microfilaremia for patients who did not cleared it. In this analysis were excluded patients with negative microfilaremia at baseline and patients with positive mff but missing value (both at baseline and at the last follow up).
4. Rates of adverse events (AE) in each treatment group.

### Statistical analysis

The analyses were performed using Epi Info version 3.5.1 (Centers for Disease Control and Prevention, Atlanta, GA, USA) and Stata vers. 15 (StataCorp. 2017. *Stata Statistical Software*: *Release 15*. College Station, TX: StataCorp LLC). Categorical variables were reported as frequencies and percentages whilst discrete or continuous variables as medians and interquartile ranges (IQR).

## Results

**Fig 1:**
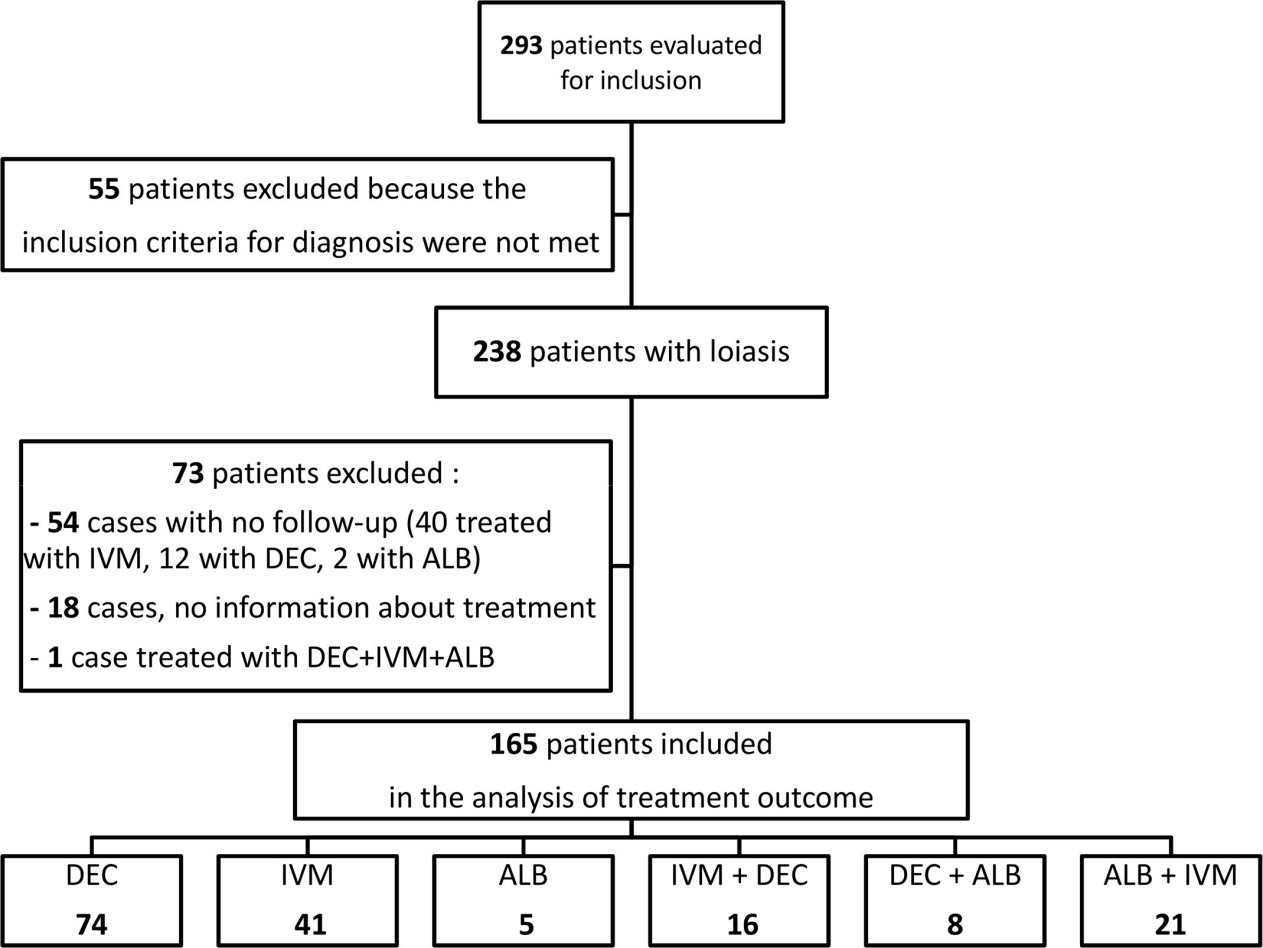
Flow chart of selection of patients with loiasis.

Eleven TropNet centers from seven countries [Belgium(1 center), Finland(1), France(1), Germany(1), Italy(3), Spain(3), and Switzerland(1)] participated in the study. Initially, 293 cases were evaluated for inclusion (Fig 1), but 55 of them did not meet the inclusion criteria for loiasis. The main results of the 238 cases of loiasis included in the study are reported in Table 1.

**Table 1:** Characteristics of the 238 patients with loiasis.

Characteristic	Number (%)	Median (Q1;Q3)
**Age**		37 (27.5; 55)
**Male gender**	129 (54.2)	
**Patient classification (n = 231)**		
Migrants	149 (64.5)	
Travelers	28 (12.1)	
Expatriates > 6 months	54 (23.4)	
**Country of diagnosis**		
France	108 (45.4)	
Belgium	71 (29.8)	
Spain	30 (12.6)	
Italy	23 (9.6)	
Germany	3 (1.3)	
Switzerland	3 (1.3)	
**Country of infection (n = 234)**		
Cameroon	133 (56.8)	
Equatorial Guinea	28 (12.0)	
Gabon	22 (9.4)	
Democratic Republic of Congo	22 (9.4)	
Congo	19 (8.2)	
Central African Republic	4 (1.7)	
Nigeria	3 (1.3)	
South Sudan	1 (0.4)	
Chad	1 (0.4)	
Angola	1 (0.4)	
**Presence of symptoms of loiasis**	234/238 (98.3)	
Calabar swellings	106 (45.3)	
Eyeworm	57 (24.2)	
Eyeworm and Calabar swelling	24 (10.3)	
**Presence of eosinophilia (>450 eos / mL)**	209 (87.8)	
**Median value of eosinophilia (mmf/mL)**		1,480 (925; 3,180)
**Presence of mf/ mL**	105 (44.3)	
**Median value of mmf**		311 (52; 1,782)

IQR = Interquartile range.

The different regimens were administered according to the schedules indicated in methods. Complete treatment and follow-up data were available for 165 cases, who were therefore included in the outcome analysis (Table 2).

**Table 2:** Number and type of patients, drug regimen for each TropNet center.

TropNet Center	N. of patients	Exp.	Mig.	Trav.	DEC	IVM	ALB	IVM + DEC	DEC + ALB	ALB + IVM
**Antwerp**	69	30	28	11	53	1		15		
**Paris** **(for France)**	43	3	31	8	4	38				1
**Negrar Verona**	19	8	8	3						19
**Barcelona** **(Vall d'Hebron)**	14		14		10		1		3	
**Madrid (Alcalà de Henares)**	11		11		4	1	1	1	4	
**Basel**	3	1	2		1		2			
**Turin**	3		2	1	1		1		1	
**Hamburg**	2	1	1		1	1				
**Florence**	1			1						1

The treatment most frequent administered was DEC (74/165, 45.1%), followed by IVM (41/165, 25%), and ALB (5/165, 3.7%). Combination therapies were: ALB + IVM in 21 out of 164 (11.6%) cases, IVM + DEC in 16 of 165 (9.7%) cases, and ALB + DEC in 8 of 165 (4.9%) cases. Baseline characteristics per administered treatment are reported in Table 3.

**Table 3:** Baseline characteristics of the patients according to the treatment group.

	DEC	IVM	ALB	IVM + DEC	DEC + ALB	ALB + IVM
Migrants	40/74 (54%)	29/40 (72.5%)	5/5 (100%)	7/16 (43.7%)	7/8 (87.5%)	9/21 (42.9%)
Patients with microfilaremia	45/74 (60.8%)	4/41 (9.8%)	5/5 (100%)	6/16 (37.5%)	8/8 (100%)	15/21 (71.4%)
Microfilaremia (per ml)–median(Q1;Q3)	309 (48–2016)	4,820 (800–7,760)	2 (1–505)	8,107 (682–16,773)	556 (145–1,300)	172 (26–791)
Eosinophilia (per ml)–median (Q1;Q3)	1,410 (860–2940)	1,406 (995–2,746)	1,450 (1,011–1,603)	4,700 (1,920–8,045)	1,600 (850–3,745)	1,620 (790–4,100)

DEC = Diethylcarbamazine, IVM = ivermectin, ALB = albendazole, IQR = Interquartile range

Migrants accounted for 42.9% in the ALB + IVM group, 43.7% in the IVM + DEC group, 54.0% in the DEC group, and 72.5% in the IVM group. Median baseline values of eosinophilia ranged between 1,406/ml and 1,620/ml in all groups, but the IVM + DEC group, in which the median eosinophilia was 4,700/ml (IQR 1,920; 8,045). Microfilaremic patients accounted for 9.8% in the IVM group, 37.5% in the IVM + DEC group, 60.8% in the DEC group, and 71.4% in the ALB + IVM group. The median values of microfilaremia were 172 mff/ml in the ALB + IVM group, 309/ml in the DEC group, 4,820/ml in the IVM group (only 4 patients), 8,107/ml in the IVM + DEC group. The median time from treatment completion to the last follow up visit was 4.5 months (IQR 2.5; 10.5). Clinical outcome is reported in Table 4.

**Table 4:** Parasitological and clinical outcome of 165 patients with follow-up.

PARASITOLOGICAL OUTCOME	DEC	IVM	ALB	IVM + DEC	DEC + ALB	ALB + IVM
Clearance of mmf + normal eosinophil count	37/74 (50.0%)	7/39 (17.9%)	0/5 (0%)	7/16 (43.7%)	3/8 (37.5%)	14/21 (66.7%)
Persisting mmf and/or eosinophilia	37/74 (50.0%)	32/39 (82.1%)	5/5 (100%)	9/16 (56.3%)	5/8 (62.5%)	7/21 (33.3%)
**CLINICAL OUTCOME**						
Clearance of symptoms	33/48 (68.7%)	13/25 (52.0%)	1/2 (50.0%)	10/14 (71.4%)	2/2 (100%)	14/15 (93.3%)
Re-occurrence of symptoms	15/48 (31.3%)	12/25 (48.0%)	1/2 (50.0%)	4/14 (28.6%)	0/2 (0%)	1/15 (6.7%)

DEC = Diethylcarbamazine, IVM = ivermectin, ALB = albendazole

Clearance of parasitemia and normal eosinophil count were observed in 67% of patients treated with ALB+IVM, in half of the patients in the DEC group, 44% in the DEC+IVM group, 38% in DEC+ALB group, 18% in the patients treated with IVM alone, in none of the 5 patients treated with ALB alone.

For two patients treated with IVM, the parasitological follow up data were unavailable. The information on clinical outcome was lacking in 58 out of 165 (35.1%). Re-occurrence of symptoms was observed in 1/15 (7%) of ALB + IVM treated cases, compared with 4/14 (29%) of IVM + DEC, 15/48 (31%) of DEC and 12/25 (48%) of IVM.

**Fig 2:**
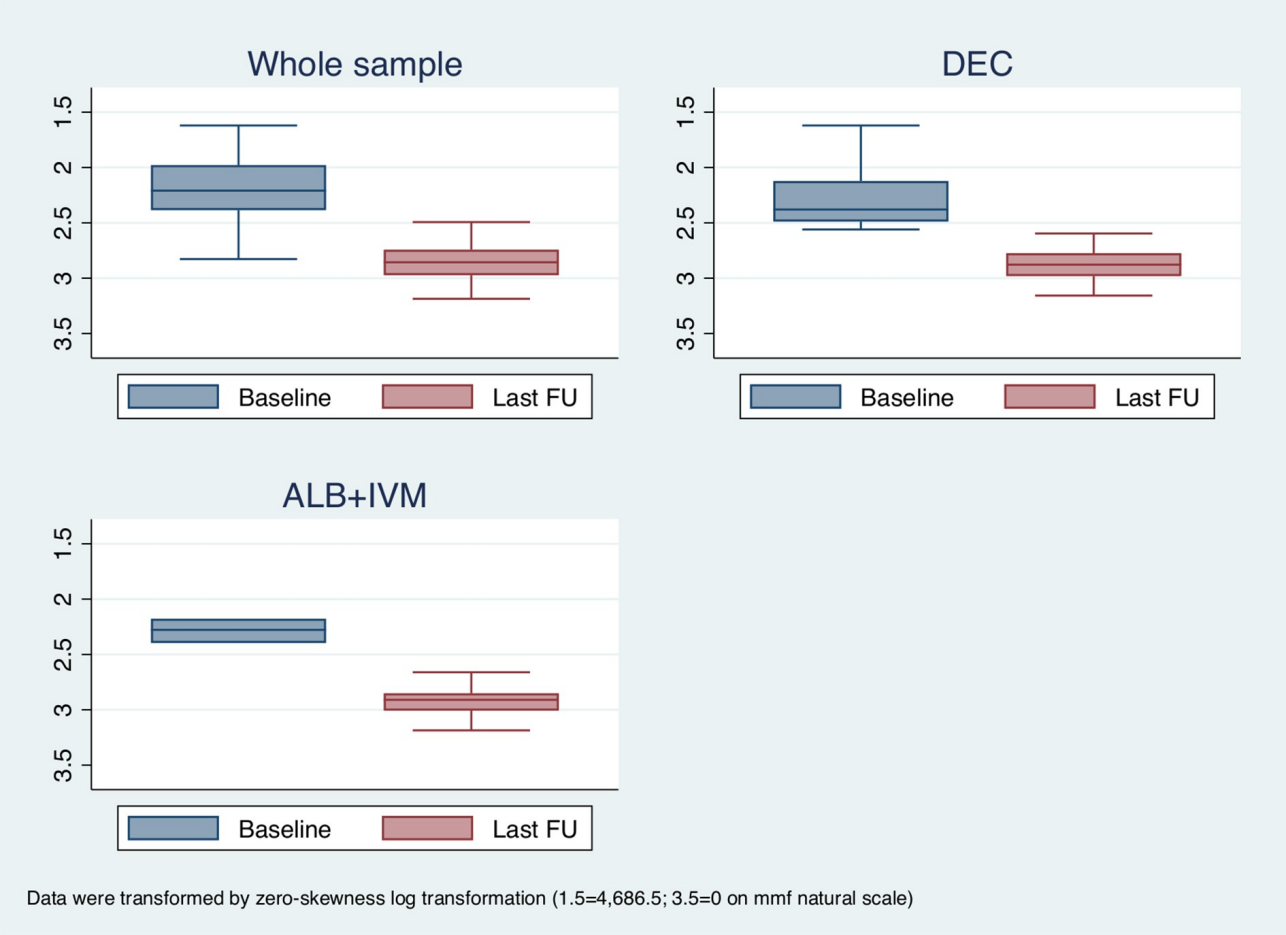
Box plots showing the trend of microfilaremia in patients who did not clear parasitemia. Due to a strong asymmetry of mmf count, variable data were transformed by zero-skewness log transformation.

We then analyzed the trend of microfilaremia in patients who did not clear parasitemia. This analysis was performed for the whole sample and for patients who were treated with either DEC or ALB+IVM, as the latter two groups were the ones that presented a sufficient number of cases permitting this analysis (excluding IVM alone as clearly ineffective). The results are shown in Fig 2: all the three groups considered demonstrated a consistent reduction in the median values of microfilaremia.

### Side effects

No serious AE was registered. Patients treated with I and A alone had no AE. Two patients in the AI group (2/21, 9.5%) reported itching; other two patients in the DA group reported itching (2/8, 25%), and one patient in the same group reported dizziness. Itching was also reported by three patients of 16 in the DI group (3/16, 18.7%), and one patient in the same group reported dyspnea. The number of AE registered for patients in the D group was 19 (19/74, 25.7%): 8 patients reported itching (8/74, 10.8%), 5 fever (5/74, 6.7%), 2 had a syncope (2/74, 2.7%), and single patients reported one of the following symptoms: thoracic pain, abdominal pain, dizziness, skin rash. None of the patients had to stop the treatment due to AE.

## Discussion

To our knowledge, this is the largest case series of loiasis treatment of imported cases ever published in non-endemic settings, where the observation of treatment response is not hampered by possible re-infections. The first striking observation was that the management of loiasis substantially differs even across specialized travel clinics of TropNet network sites. Not only six different therapeutic regimens were used, but also the timing and lab approach for follow up differed. These discrepancies could be due to different local protocols as well as to (un)availability of the drugs (IVM is not registered in the majority of the European countries, DEC is not registered and often very difficult to find) and peculiarities of the patients. Indeed, 85.4% of the patients were either migrants or expatriates, who are not sedentary and could be more difficult to follow up at regular time points.

In terms of outcome, we found that only 50% of patients treated with DEC alone achieved a parasitological cure. This drug showed a cure rate of 73% in a work by Saito et al[19], while in the only study reporting a long-term follow up after DEC treatment in non-endemic countries the efficacy after a single treatment course resulted of only 38%[5]. Taken all together, these results suggest that DEC monotherapy is often unsuccessful, despite its micro and macrofilaricidal activity[19]. The combination of DEC with IVM or ALB did not improve the proportion of parasitological cure. On the other hand, the combination ALB + IVM provided a high proportion of parasitological cure. Although this observation should be taken with caution, given the small number on patients in this group, exploring the efficacy of this combination deserves further research, considering: 1) the synergy of the mode of action of the two drugs (ALB probable macrofilaricidal and IVM microfilaricidal); 2) safety issues: given as the first drug, ALB would reduce the levels of mmf and overcome the possibility of severe adverse events due to a massive death of mff, caused by IVM; 3) the serious threaten to the availability of DEC. Indeed, this treatment option also fits with the treatment strategy previously proposed by Boussinesq[6]. However, larger prospective studies to explore whether this regimen could represent a valid alternative to DEC are needed. In addition, it must be taken into account that in our work almost all patients (20/21) treated with this regimen received ALB at the dose of 400 mg twice a day for 28 days, which is a higher dose given for a longer period compared with what has been previously reported[13]. IVM alone showed poor efficacy (parasitological cure 18%), which is not surprising as its action seems to be purely microfilaricidal. Although the drug is not capable of clearing the infection, it can contribute to reduce transmission in endemic countries by reducing microfilaremia in affected populations.

### Study limitations

The main limitation of this study is represented by its retrospective design and the substantial proportion of missing data. Also, different drug dosages and durations were used in the study sites, thus limiting the comparison among the regimens. A statistical inference on the different outcomes after each regimen was not performed, as the recruitment was not random and the groups differed markedly before treatment. Indeed, clearance of symptoms, reduction of microfilaremia and eosinophilia are the parameters usually considered to follow up patients with *Loa loa* infection, but there are no clear indications for the timing of follow-up and the cut-off values defining cure. Finally, the clinical outcome may be difficult to assess, for example in case of persistence of symptoms not clearly attributable to loiasis (such as edemas reported by the patients, but not observed by the health staff). Despite these limitations, we were able to provide useful clinical information on a neglected disease for which complete follow up data are hardly available[16–19].

## Conclusions

This paper shows that, in absence of specific guidelines, different reference centers for tropical diseases in Europe use different treatment schedules for loiasis. Our study suggests that some alternative recommendations may be possible for the treatment of loiasis in non-endemic areas, also in consideration of the unavailability of some drugs (namely, DEC). Ideally, a randomized clinical trial would provide a much more robust base of evidence to support management guidelines, but its feasibility in non-endemic countries is questionable. Nevertheless, an observational, prospective study, with well-defined criteria for inclusion and for definition of cure, would be a valuable option in order to evaluate the proposed management indications.

## Supporting information

S1 Checklist STROBE checklist. (DOCX)

## References

[1] Zoure HG, Wanji S, Noma M, Amazigo UV, Diggle PJ, et al (2011) The geographic distribution of Loa loa in Africa: results of large-scale implementation of the Rapid Assessment Procedure for Loiasis (RAPLOA). PLoS Negl Trop Dis 5: e1210 10.1371/journal.pntd.0001210 21738809 PMC3125145

[2] Boussinesq M (2006) Loiasis. Ann Trop Med Parasitol 100: 715–731. 10.1179/136485906X112194 17227650

[3] Chesnais CB, Takougang I, Paguele M, Pion SD, Boussinesq M (2017) Excess mortality associated with loiasis: a retrospective population-based cohort study. Lancet Infect Dis 17: 108–116. 10.1016/S1473-3099(16)30405-4 27777031

[4] Metzger WG, Mordmuller B (2013) Loa loa-does it deserve to be neglected? Lancet Infect Dis. 10.1016/S1473-3099(13)70263-9 24332895

[5] Klion AD, Ottesen EA, Nutman TB (1994) Effectiveness of diethylcarbamazine in treating loiasis acquired by expatriate visitors to endemic regions: long-term follow-up. J Infect Dis 169: 604–610. 8158033 10.1093/infdis/169.3.604

[6] Boussinesq M (2012) Loiasis: new epidemiologic insights and proposed treatment strategy. J Travel Med 19: 140–143. 10.1111/j.1708-8305.2012.00605.x 22530819

[7] Buonfrate D, Gobbi F, Bisoffi Z (2017) Helminths in organ transplantation. Lancet Infect Dis 17: 581–582. 10.1016/S1473-3099(17)30270-0 28555581

[8] http://www.tropnet.eu/indexphp?id=200&fsize=2%27A%3D0.

[9] Gardon J, Kamgno J, Folefack G, Gardon-Wendel N, Bouchite B, et al (1997) Marked decrease in Loa loa microfilaraemia six and twelve months after a single dose of ivermectin. Trans R Soc Trop Med Hyg 91: 593–594. 9463678 10.1016/s0035-9203(97)90041-9

[10] Gardon J, Gardon-Wendel N, Demanga N, Kamgno J, Chippaux JP, et al (1997) Serious reactions after mass treatment of onchocerciasis with ivermectin in an area endemic for Loa loa infection. Lancet 350: 18–22. 10.1016/S0140-6736(96)11094-1 9217715

[11] Tabi TE, Befidi-Mengue R, Nutman TB, Horton J, Folefack A, et al (2004) Human loiasis in a Cameroonian village: a double-blind, placebo-controlled, crossover clinical trial of a three-day albendazole regimen. Am J Trop Med Hyg 71: 211–215. 15306713

[12] Kamgno J, Nguipdop-Djomo P, Gounoue R, Tejiokem M, Kuesel AC (2016) Effect of Two or Six Doses 800 mg of Albendazole Every Two Months on Loa loa Microfilaraemia: A Double Blind, Randomized, Placebo-Controlled Trial. PLoS Negl Trop Dis 10: e0004492 10.1371/journal.pntd.0004492 26967331 PMC4788450

[13] Klion AD, Horton J, Nutman TB (1999) Albendazole therapy for loiasis refractory to diethylcarbamazine treatment. Clin Infect Dis 29: 680–682. 10530467 10.1086/598654

[14] Nutman TB, Miller KD, Mulligan M, Ottesen EA (1986) Loa loa infection in temporary residents of endemic regions: recognition of a hyperresponsive syndrome with characteristic clinical manifestations. J Infect Dis 154: 10–18. 3458832 10.1093/infdis/154.1.10

[15] Churchill DR, Morris C, Fakoya A, Wright SG, Davidson RN (1996) Clinical and laboratory features of patients with loiasis (Loa loa filariasis) in the U.K. J Infect 33: 103–109. 8889997 10.1016/s0163-4453(96)93005-4

[16] Gantois N, Rapp C, Gautret P, Ficko C, Savini H, et al (2013) Imported loiasis in France: a retrospective analysis of 47 cases. Travel Med Infect Dis 11: 366–373. 10.1016/j.tmaid.2013.08.005 24035648

[17] Antinori S, Schifanella L, Million M, Galimberti L, Ferraris L, et al (2012) Imported Loa loa filariasis: three cases and a review of cases reported in non-endemic countries in the past 25 years. Int J Infect Dis 16: e649–662. 10.1016/j.ijid.2012.05.1023 22784545

[18] Gobbi F, Postiglione C, Angheben A, Marocco S, Monteiro G, et al (2014) Imported loiasis in Italy: an analysis of 100 cases. Travel Med Infect Dis 12: 713–717. 10.1016/j.tmaid.2014.07.004 25131142

[19] Saito M, Armstrong M, Boadi S, Lowe P, Chiodini PL, et al (2015) Clinical Features of Imported Loiasis: A Case Series from the Hospital for Tropical Diseases, London. Am J Trop Med Hyg 93: 607–611. 10.4269/ajtmh.15-0214 26101271 PMC4559705

